# Supplementary material for: Functional Signatures in Non-Small-Cell Lung Cancer: A Systematic Review and Meta-Analysis of Sex-Based Differences in Transcriptomic Studies
Source: Cancers (Basel). 2021 Jan 5;13(1):143. doi: 10.3390/cancers13010143 (PMC7796260; doi:10.3390/cancers13010143)
Supplement: Supplementary file 1 [file cancers-13-00143-s001.zip › supplementary/SupplementaryFigureS2.pdf]

Supplementary Figure S2. Prognostic effect of transcriptional pathways. Overall survival differences between low and high expression groups, in men (left) and women (right). Low and high groups include under or overexpressed genes respectively, related to each pathway. TCGA and GEO datasets, which contain expression levels and survival information, were used from Kaplan-Meier Plotter web tool (https://kmplot.com/).

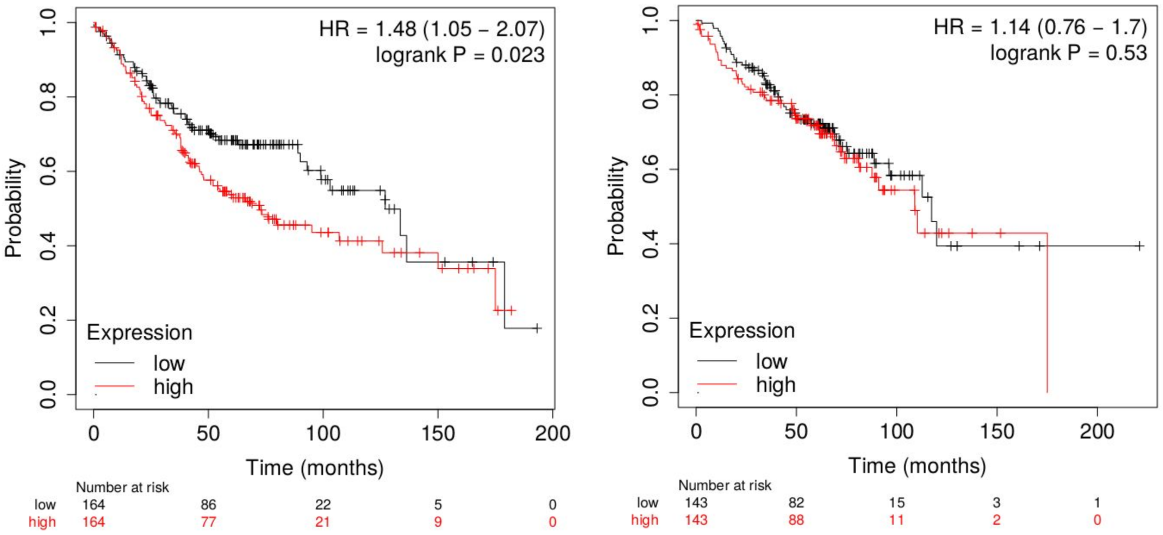

A. Overall survival sex differences between low and high expression groups, in genes related to "negative regulation of leukocyte degranulation (GO:0043301)".

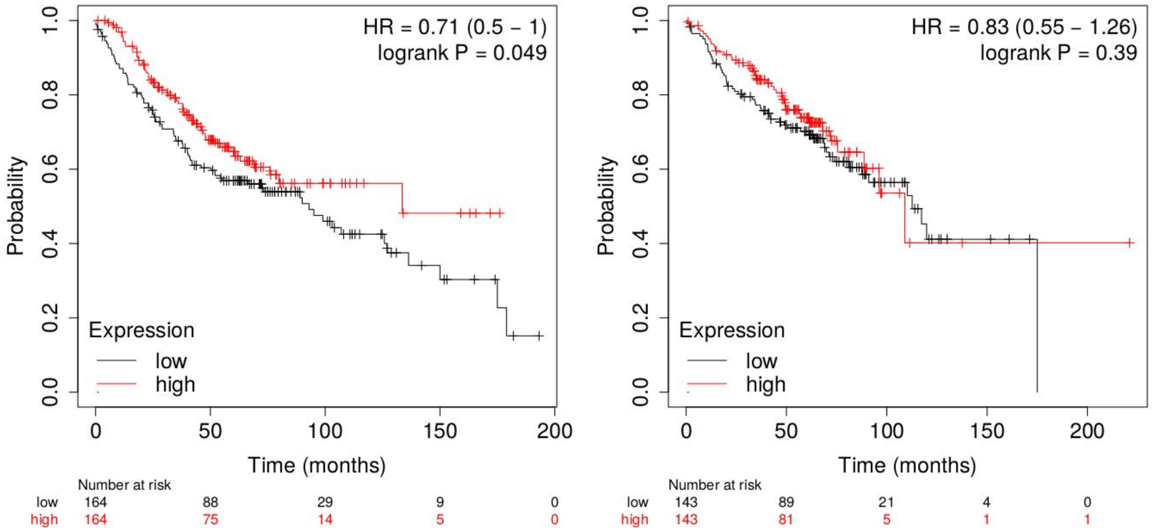

B. Overall survival sex differences between low and high expression groups, in genes related to "inositol trisphosphate metabolic process (GO:0032957)".

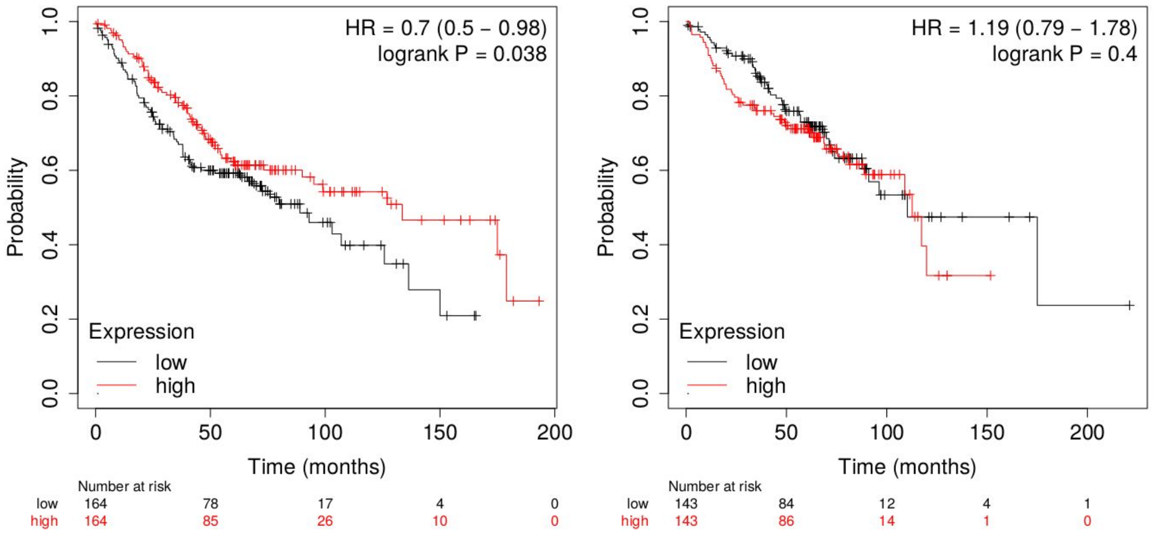

C. Overall survival sex differences between low and high expression groups, in genes related to “negative regulation of chromatin silencing (GO:0031936)”.

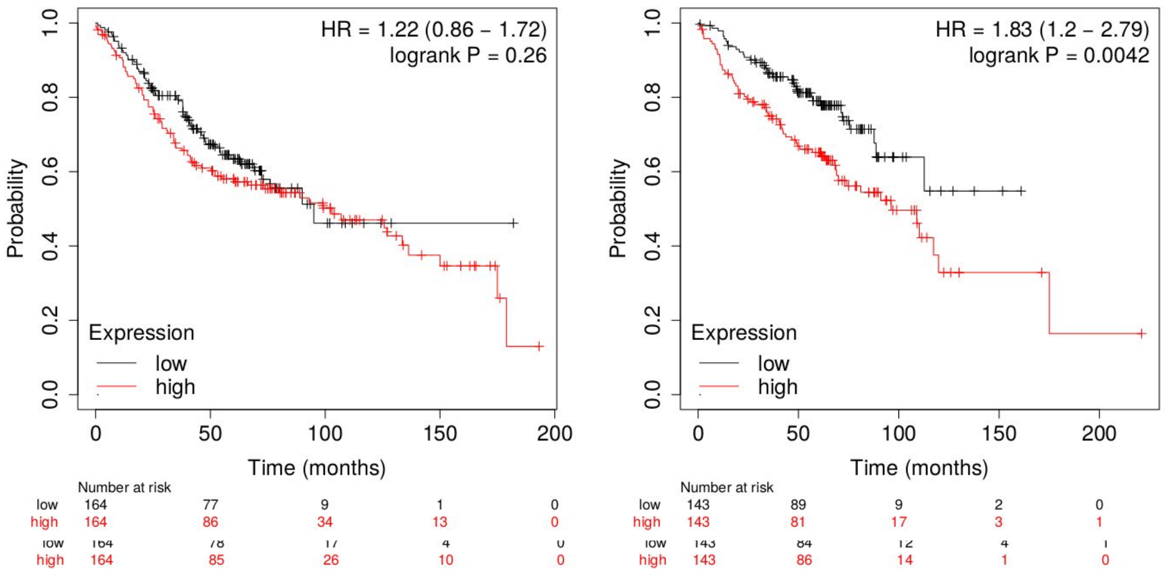

D. Overall survival sex differences between low and high expression groups, in genes related to “inner ear auditory receptor cell differentiation (GO:0000491)”.

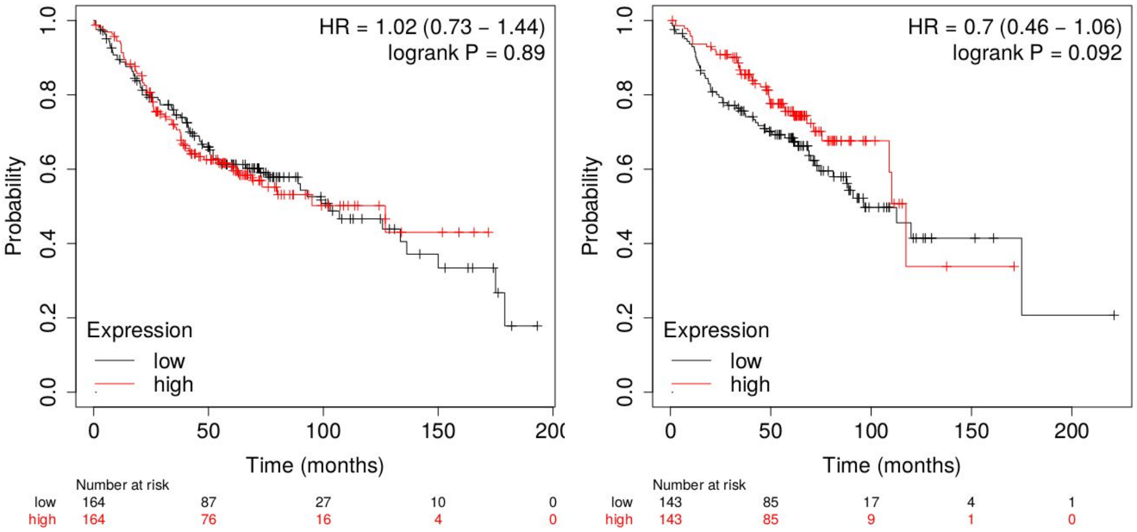

E. Overall survival sex differences between low and high expression groups, in genes related to "positive regulation of leukocyte adhesion to vascular endothelial cells (GO:1904996)".
