# Supplementary material for: Functional Signatures in Non-Small-Cell Lung Cancer: A Systematic Review and Meta-Analysis of Sex-Based Differences in Transcriptomic Studies
Source: Cancers (Basel). 2021 Jan 5;13(1):143. doi: 10.3390/cancers13010143 (PMC7796260; doi:10.3390/cancers13010143)
Supplement: Supplementary file 1 [file cancers-13-00143-s001.zip › supplementary/SupplementaryTableS1.docx]

**Table S1.** Distribution of the clinicopathological characteristics of each study population.

| **Study** | **Samples** | **Age**  **(median + SD)** | **Sex (%)** | **Smoking Status (%)** | **Histology (%)** | **Stage (%) *** | **Mutations and Fusions (%) *** |
| --- | --- | --- | --- | --- | --- | --- | --- |
| GSE10072 | 80 | 68 ± 6.68 | Men 59 (73.75%)  Women 21 (26.25%) | Non-smoker 19 (23.75%)  Smoker 61 (76.25%) | Adenocarcinoma 43 (53.75%)  Control 37 (46.25%) | IA 5 (1.63%)  IB 17 (39.53%)  IIA 3 (6.98%)  IIB 18 (41.86%) | Not reported |
| GSE19188 | 84 | 65.07 ± 10.39 | Men 62 (73.81%)  Women 22 (26.19%) | Non-smoker 50 (59.52%)  Smoker 34 (40.47%) | Adenocarcinoma 32 (38.10%)  Control 52 (61.90%) | IA 10 (31.25%)  IB 15 (46.88%)  IIB 7 (21.87%) | Not reported |
| GSE31210 | 246 | 61 ± 8.08 | Men 116 (47.15%)  Women 130 (52.85%) | Non-smoker 123 (50.00%)  Smoker 123 (50.00%) | Adenocarcinoma 226 (91.87%)  Control 20 (8.13%) | IA 114 (50.44%)  IB 54 (23.89%)  IIA 58 (25.67%) | ALK 11 (4.87%)  EGFR 127 (56.19%)  KRAS 20 (8.85%)  Wild-type 68 (30.09%) |
| GSE32863 | 90 | 72 ± 9.33 | Men 20 (22.22%)  Women 70 (77.78%) | Non-smoker 47 (52.22%)  Smoker 43 (47.78%) | Adenocarcinoma 45 (50.00%)  Control 45 (50.00%) | IA 16 (35.56%)  IB 18 (40.00%)  IIA 9 (20.00%)  IIB 2 (4.44%) | EGFR 12 (26.67%)  KRAS 15 (33.33%)  LKB1 4 (8.89%)  KRAS + LKB1 2 (4.44%)  Wild-type 12 (26.67%) |
| GSE63459 | 63 | 64 ± 11.37 | Men 29 (46.03%)  Women 34 (53.97%) | Non-smoker 8 (12.70%)  Smoker 55 (87.30%) | Adenocarcinoma 32 (50.79%)  Control 31 (49.21%) | I 32 (100.00%) | KRAS 1 (3.125%)  KRAS + tp53 2 (6.25%)  tp53 10 (31.25%)  Unknown 5 (15.63%)  Wild-type 14 (43.75%) |
| GSE75037 | 154 | 70 ± 9.73 | Men 46 (29.87%)  Women 108 (70.13%) | Non-smoker 55 (35.71%)  Smoker 99 (64.29%) | Adenocarcinoma 71 (46.11%)  Control 83 (53.89%) | IA 25 (35.21%)  IB 26 (36.62%)  IIA 3 (4.23%)  IIB 17 (23.94%) | EGFR 16 (22.54%)  KRAS 25 (35.21%)  LKB1 6 (8.45%)  KRAS + LKB1 5 (7.04%)  Wild-type 19 (26.76%) |
| GSE81089 | 100 | 67.5 ± 7.37 | Men 39 (39.00%)  Women 61 (61.00%) | Non-smoker 8 (8.00%)  Smoker 92 (92.00%) | Adenocarcinoma 81 (81.00%)  Control 19 (19.00%) | IA 44 (54.32%)  IB 18 (22.22%)  IIA 8 (9.88%)  IIB 11 (13.58%) | Not reported |
| GSE87340 | 54 | 67 ± 12.44 | Men 8 (14.81%)  Women 46 (85.19%) | Non-smoker 54 (100.00%)  Smoker 0 (0.00%) | Adenocarcinoma 27 (50.00%)  Control 27 (50.00%) | IA 10 (37.04%)  IB 17 (62.96%) | Not reported |
| TCGA | 458 | 65.79 ± 9.93 | Men 209 (45.63%)  Women 249 (54.37%) | Non-smoker 132 (28.82%)  Smoker 326 (71.18%) | Adenocarcinoma 415 (90.61%)  Control 43 (9.39%) | I 5 (1.20%)  IA 136 (32.77%)  IB 151 (36.39%)  II 1 (0.24%)  IIA 50 (12.05%)  IIB 72 (17.35%) | Not reported |

Only four of the nine studies selected that met the inclusion criteria possessed available information regarding disease-relevant mutations. Thus, we do not include the mutation status of genes such as ALK or EGFR in the study. *Percentage calculated excluding control samples.
