# Supplementary material for: Functional Signatures in Non-Small-Cell Lung Cancer: A Systematic Review and Meta-Analysis of Sex-Based Differences in Transcriptomic Studies
Source: Cancers (Basel). 2021 Jan 5;13(1):143. doi: 10.3390/cancers13010143 (PMC7796260; doi:10.3390/cancers13010143)
Supplement: Supplementary file 1 [file cancers-13-00143-s001.zip › supplementary/SupplementaryTableS2.docx]

**Table S2.** Summary of differential expression analysis results. Two exploratory differential expression analyses were performed (ADC Women - Control Women, ADC.W -Control.W; ADC Men - Control Men, ADC.M - Control.M), together with the contrast of interest: (ADC.W - Control.W) - (ADC.M - Control.M). When performing the contrast of interest, “Up” terms are overrepresented in female lung adenocarcinoma patients, while “Down” terms are overrepresented in male lung adenocarcinoma patients.

| **Study** |  | **(ADC.W-ControlW)-(ADC.M-Control.M)** | **ADC.W-ControlW** | **ADC.M-Control.M** |
| --- | --- | --- | --- | --- |
| GSE10072 | Up | 0 | 1199 | 3182 |
|  | Down | 0 | 1296 | 2688 |
| GSE19188 | Up | 0 | 2348 | 5828 |
|  | Down | 0 | 2111 | 3830 |
| GSE31210 | Up | 0 | 3310 | 3560 |
|  | Down | 0 | 2370 | 2543 |
| GSE32863 | Up | 6 | 5243 | 2458 |
|  | Down | 1 | 4507 | 2172 |
| GSE63459 | Up | 0 | 2409 | 1561 |
|  | Down | 0 | 2064 | 1611 |
| GSE75037 | Up | 1 | 5779 | 4115 |
|  | Down | 1 | 5117 | 3680 |
| GSE81089 | Up | 3 | 2654 | 3564 |
|  | Down | 0 | 3383 | 4262 |
| GSE87340 | Up | 1 | 4887 | 1875 |
|  | Down | 3 | 4958 | 1841 |
| TCGA | Up | 1 | 5861 | 5397 |
|  | Down | 0 | 5569 | 5269 |
