# Supplementary material for: Functional Signatures in Non-Small-Cell Lung Cancer: A Systematic Review and Meta-Analysis of Sex-Based Differences in Transcriptomic Studies
Source: Cancers (Basel). 2021 Jan 5;13(1):143. doi: 10.3390/cancers13010143 (PMC7796260; doi:10.3390/cancers13010143)
Supplement: Supplementary file 1 [file cancers-13-00143-s001.zip › supplementary/SupplementaryTableS3.docx]

**Table S3.** Genes differentially expressed between male and female lung adenocarcinoma patients.

| **ENTREZ ID** | **Gene Name** | **Up / Down** | **logFC** | **adj.pval** | **Study** |
| --- | --- | --- | --- | --- | --- |
| 9086 | Eukaryotic translation initiation factor 1A Y-linked | Up | 0.606  1.514 | 0.014  7.38*10-7 | GSE32863  GSE75037 |
| 146330 | F-box and leucine rich repeat protein 16 | Down | 1.247  2.157 | 0.028  0.003 | GSE32863  GSE75037 |
| 3394 | interferon regulatory factor 8 | Up | 1.177 | 0.028 | GSE32863 |
| 80301 | pleckstrin homology domain containing O2 | Up | 0.986 | 0.028 | GSE32863 |
| 3689 | integrin subunit beta 2 | Up | 1.393 | 0.043 | GSE32863 |
| 11309 | solute carrier organic anion transporter family member 2B1 | Up | 1.123 | 0.049 | GSE32863 |
| 83706 | fermitin family member 3 | Up | 0.866 | 0.049 | GSE32863 |
| 252948 | testis-specific transcript, Y-linked 16 | Up | 1.655  2.983 | 1.98*10-7  3.3*10-14 | GSE81089  TCGA |
| 107987337 | ZFY antisense RNA 1 | Up | 1.609 | 0.001 | GSE81089 |
| 6736 | sex determining region Y | Up | 1.519 | 0.007 | GSE81089 |
| 694 | BTG anti-proliferation factor 1 | Up | 0.859 | 0.007 | GSE87340 |
| 64582 | G protein-coupled receptor 135 | Down | 2.213 | 0.007 | GSE87340 |
| 22979 | EFR3 homolog B | Down | 1.799 | 0.03 | GSE87340 |
| 54753 | zinc finger protein 853 | Down | 1.581 | 0.04 | GSE87340 |

logFC: logarithm of the fold change of the expression between the compared groups, adj.pval: adjusted FDR p-value.
