# Supplementary material for: Functional Signatures in Non-Small-Cell Lung Cancer: A Systematic Review and Meta-Analysis of Sex-Based Differences in Transcriptomic Studies
Source: Cancers (Basel). 2021 Jan 5;13(1):143. doi: 10.3390/cancers13010143 (PMC7796260; doi:10.3390/cancers13010143)
Supplement: Supplementary file 1 [file cancers-13-00143-s001.zip › supplementary/SupplementaryTableS6.docx]

**Table S6.** Software and versions used in this study.

| **Software / R package** | **Version** |
| --- | --- |
| R | 3.5.3 |
| AnnotationDbi | 1.44.0 |
| Biobase | 2.42.0 |
| biomaRt | 2.38.0 |
| edgeR | 3.24.3 |
| GEOQuery | 2.50.5 |
| ggdendro | 0.1-20 |
| ggpubr | 0.2 |
| hgu133plus2.db | 3.2.3 |
| illuminaHumanv3.db | 1.26.0 |
| KEGG.db | 3.2.3 |
| limma | 3.38.3 |
| mdgsa | 1.14.0 |
| metafor | 2.1-0 |
| methods | 3.5.3 |
| org.Hs.eg.db | 3.7.0 |
| reshape | 0.8.8 |
| stats | 3.5.3 |
| SummarizedExperiment | 1.12.0 |
| TCGAbiolinks | 2.10.5 |
| tidyverse | 1.2.1 |
| UpSetR | 1.3.3 |
| utils | 3.5.3 |

The **Metafun-NSCLC** web tool is freely available at https://bioinfo.cipf.es/metafun-nsclc
