# Supplementary figures and images for: Functional Signatures in Non-Small-Cell Lung Cancer: A Systematic Review and Meta-Analysis of Sex-Based Differences in Transcriptomic Studies
Source: Cancers (Basel). 2021 Jan 5;13(1):143. doi: 10.3390/cancers13010143 (PMC7796260; doi:10.3390/cancers13010143)

**Supplementary Figure S1**. Information regarding sex distribution among reviewed studies.


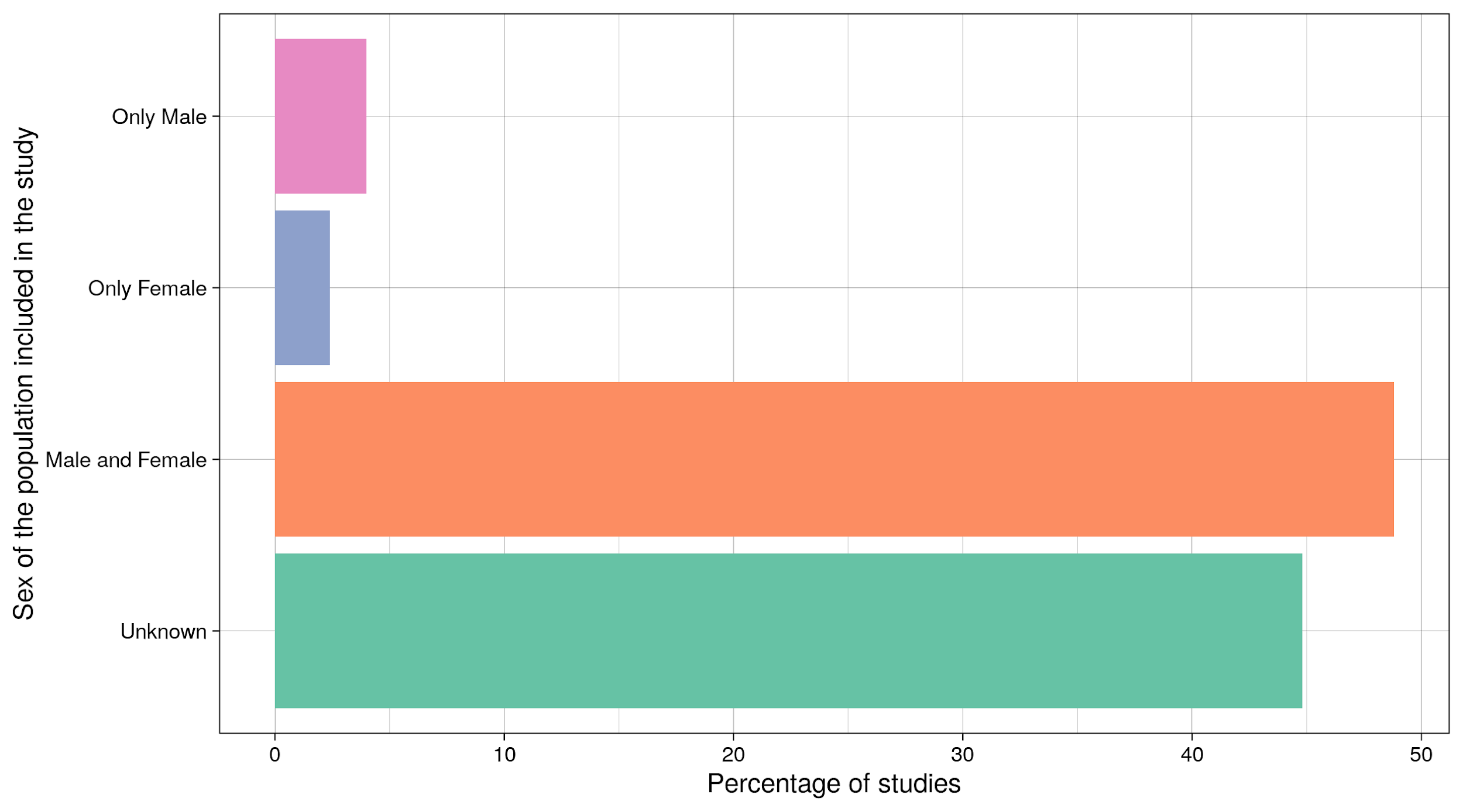

Supplement: Supplementary file 1 [file cancers-13-00143-s001.zip › supplementary/SupplementaryFigureS1.docx]

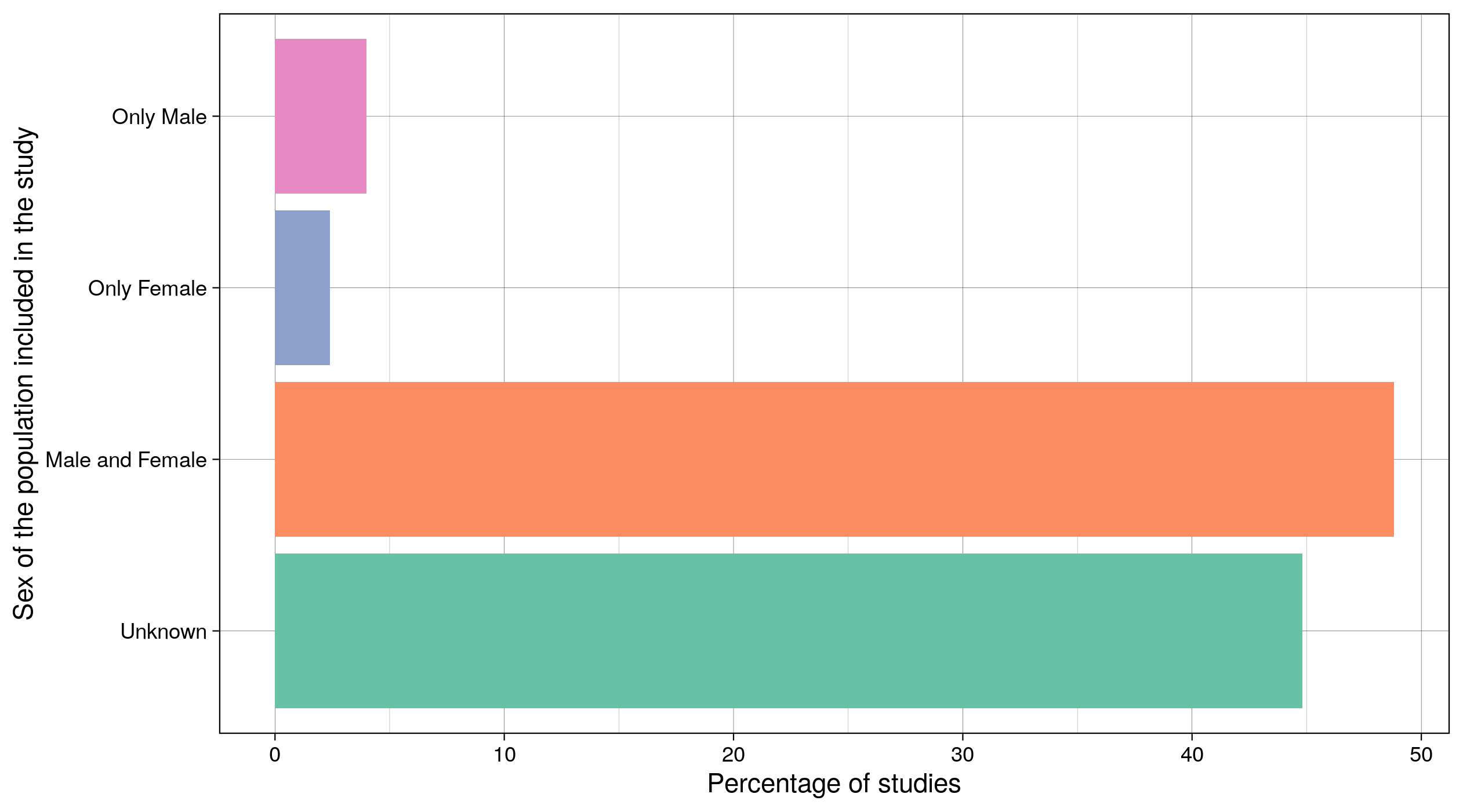

Supplement: Supplementary file 1 [file cancers-13-00143-s001.zip › supplementary/SupplementaryFigureS1.png]
